# From next-generation sequencing to systematic modeling of the gut microbiome

**DOI:** 10.3389/fgene.2015.00219

**Published:** 2015-06-23

**Authors:** Boyang Ji, Jens Nielsen

**Affiliations:** Division of Systems and Synthetic Biology, Department of Biology and Biological Engineering, Chalmers University of Technology, Göteborg, Sweden

**Keywords:** next-generation sequencing, gut microbiome, metabolic modeling, species interactome, systematic modeling, personalized medicine

## Abstract

Changes in the human gut microbiome are associated with altered human metabolism and health, yet the mechanisms of interactions between microbial species and human metabolism have not been clearly elucidated. Next-generation sequencing has revolutionized the human gut microbiome research, but most current applications concentrate on studying the microbial diversity of communities and have at best provided associations between specific gut bacteria and human health. However, little is known about the inner metabolic mechanisms in the gut ecosystem. Here we review recent progress in modeling the metabolic interactions of gut microbiome, with special focus on the utilization of metabolic modeling to infer host–microbe interactions and microbial species interactions. The systematic modeling of metabolic interactions could provide a predictive understanding of gut microbiome, and pave the way to synthetic microbiota design and personalized-microbiome medicine and healthcare. Finally, we discuss the integration of metabolic modeling and gut microbiome engineering, which offer a new way to explore metabolic interactions across members of the gut microbiota.

## Introduction

The human gut microbiome, represented by trillions of microorganisms colonized in the human gut, is a major contributor to human metabolism and health (Backhed et al., 2005; Turnbaugh and Gordon, 2009). The microbiota locating in the gastrointestinal tract is able to perform multiple roles for the human host, including nutritional, physiological and immunological functions, which are distinct from the host’s own constitutive resources (Guarner and Malagelada, 2003; Kovatcheva-Datchary et al., 2013). Therefore, the gut microbiome is considered as a human organ with its own specific functions and complexity (O’Hara and Shanahan, 2006; Baquero and Nombela, 2012). Historically, gut microbiome studies have been restricted due to the difficulties in culturing many of these gut microbial species in laboratory conditions (Lagier et al., 2012a). Development of next-generation sequencing (NGS) based metagenomics has enabled bypassing of the traditional culture-dependent bias and has significantly expanded our understanding of the composition, diversity and roles of the gut microbiome in human health and diseases. However, such gene/genome-centric high-throughput approaches provide little mechanistic insights into how gut microbiota interact with each other and with the host, and how these interactions contribute to the host metabolic machinery. Therefore, the shift from gene/genome-centric analysis to mechanism-centric methods by integrating omics and experimental data with existing knowledge at the system-level will be a critical next step for gut microbiome studies. Here we will review and discuss recent progresses in systematic modeling of the gut microbiome, with special focus on the application of metabolic modeling to infer host–microbe interactions and microbial species interactions.

## Next-Generation Sequencing in Gut Microbiome Research

**FIGURE 1:**
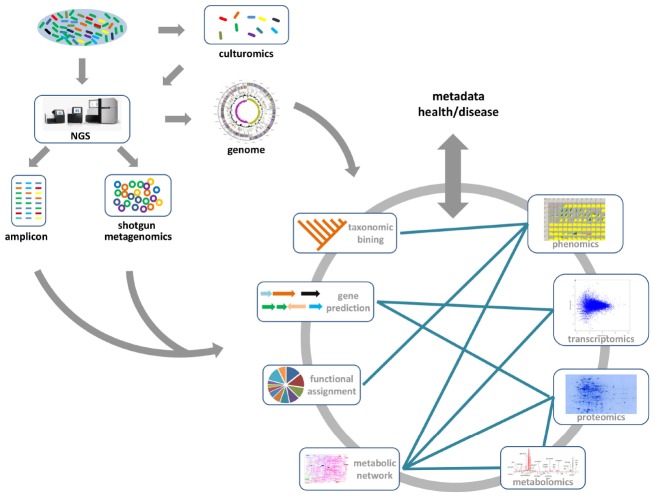
**The gene/genome-centric approach for the gut microbiome.** Generally, 16S-rRNA based amplicon sequencing and whole shotgun sequencing are the two main metagenomic approaches for gut microbiome studies. From metagenome data, the taxonomic compositions and functional categories of the gut microbial communities, which may be associated with the health or disease state, can be inferred. Moreover, the combination of culturomics and NGS methods provides deeper information about the functional roles of specific gut microbial species. Other available “omics” data (transcriptomics, proteomics, metabolomics, and phenomics) provides much deeper insight into the functional role of gut microbes in human health and disease. Integrating these data with metagenomics data, especially metabolic models reconstructed from metagenomic studies, will provide a comprehensive view of metabolic interactions between microbes and host.

DNA sequencing technology was first developed in 1975 (Sanger and Coulson, 1975), and is based on the selective incorporation of labeling chain-terminating ddNTPs by DNA polymerase during *in vitro* DNA replication. However, it was historically expensive, time-consuming and laborious for high-throughput studies. NGS technologies are based on the principle of massively parallel sequencing, which has been extensively reviewed elsewhere (Ronaghi, 2001; Mardis, 2008). The advances of NGS technology have facilitated gut microbiome research, and enabled the exploration of genetic and functional diversity of uncultured gut microbial communities with affordable costs and sufficient throughput. Amplicon-based profiling is one of the most widely used methods for characterizing gut microbiome diversity. Here, a taxonomically informative gene marker (usually 16S rRNA for bacteria and archaea), which is common for organisms to be studied, is targeted and amplified from the total DNA by PCR. The resulting amplicons are sequenced, and downstream bioinformatics analyses are performed to determine the relative taxonomical abundances in the sample (Figure 1). Comparison of Gene marker profiles across samples clarifies how microbial diversity is associated with host–microbe interactions (Qin et al., 2012), or different environmental factors, such as diet (Carmody et al., 2014; Xu and Knight, 2015), drugs (Dethlefsen et al., 2008; Dethlefsen and Relman, 2011), or probiotics (Wang et al., 2015). However, amplicon sequencing typically only resolves the taxonomic composition of the gut microbiome. It is impossible to provide direct evidence of the biological functions associated with the gut microbial community. Thus, recently developed computational approaches, such as implemented in PICRUSt (Langille et al., 2013) and Genome traits (Keller et al., 2014), were successfully employed to infer the community’s functional potential by bridging 16S rRNA gene information with reference genomes (Figure 1).

Shotgun genomic sequencing is an alternative metagenomic approach for characterizing the gut microbiome. Instead of amplification against a specific gene marker, total DNA is subsequently sequenced and analyzed. The following bioinformatics analysis usually involves gene predictions and functional annotation besides taxonomic binning (Figure 1), which provides a more global way to simultaneously explore taxonomic composition and functional capacity of the gut microbiome. Such taxonomic and functional profiles can be used to investigate the interactions between the gut microbiome and disease state or life styles. Recently, the metagenomic-wide association study of 145 European woman who had type-2 diabetes (T2D), impaired glucose tolerance, or healthy controls, also showed significant correlations of specific gut microbes (e.g., *Roseburia* species and *Faecalibacterium prausnitzii*) and their genes with T2D (Karlsson et al., 2013b). An empirical model based on the metagenomic profiles from this cohort enabled identification of women in the pre-T2D cohort who also have high levels of blood plasma markers associated with T2D. Moreover, the taxonomic markers of the gut microbiota had been identified to distinguish the colorectal carcinoma patients from tumor-free controls, which provide a non-invasive fecal readout for accurate detection of colorectal cancer (Zeller et al., 2014). In the context of individualized medicine, it will be desirable to use these biomarkers in a diagnostic or therapeutic setting.

Although NGS-based sequencing has dramatically expanded our knowledge of the gut microbiome, current culture-independent metagenomics generate mixed data to reflect community-level characteristics rather than species-specific features. Consequently, there is a renewed interest in high-throughput culture methods—culturomics (Figure 1; Greub, 2012). A recent anaerobic culturing study on a rich medium showed that ∼50% species can be identified from the cultured samples (Goodman et al., 2011). Utilizing 212 different culture conditions with mass spectrometry (MS) techniques and NGS approach, as many as 32,500 different colonies had been recovered from three stool samples (Lagier et al., 2012b). The identified gut microbiota included 174 species never described before in the human gut, and 31 new species and genera were sequenced, generating ∼10,000 previously unknown genes (Lagier et al., 2012b). Moreover, the representative gut microbiota species with antibiotic resistance had been successfully cultivated from fecal samples by combining novel culture conditions and rapid phenotypic profiling (Rettedal et al., 2014). For unculturable microorganisms, single cell genomics have been introduced to investigate uncultivated species from a broad range of ecosystems (Figure 1; Lasken, 2012; Blainey and Quake, 2014). As single-cell genomics need a step for amplifying the genome from a single cell, such an approach has the potential to speed up the discovery of new species without prior cultivation. Application of single-cell sequencing to two species of the bee gut microbiota: *Gilliamella apicola* and *Snodgrassella alvi*, revealed extensive variations in intraspecific divergence of protein-encoding genes (Engel et al., 2014). Beyond the metagenome and single-cell genome, numerous transcriptomics, proteomics, metabolomics, and phenomics data are becoming available for gut microbiome studies (Figure 1). The integrative analysis of these omics data will be important for understanding the intrinsic mechanism of host–microbiome interaction.

## Metabolic Modeling of the Human Gut Microbiome

Even though our understanding of the gut microbiome has advanced rapidly with NGS, genomic sequencing based analysis is not sufficient to decipher the mechanisms of how the microbiome affects human health. It is necessary to infer the metabolic activities of the gut microbiota and quantify the metabolic interactions between the gut microbes, and the interaction between microbes and host, which will then provide insight into the molecular mechanisms of gut microbiome contributing to human health. In this context, a modeling based approach will be an effective way to study the gut microbial metabolic interactions at the systems level (Karlsson et al., 2011; Manor et al., 2014; Shoaie and Nielsen, 2014).

**FIGURE 2:**
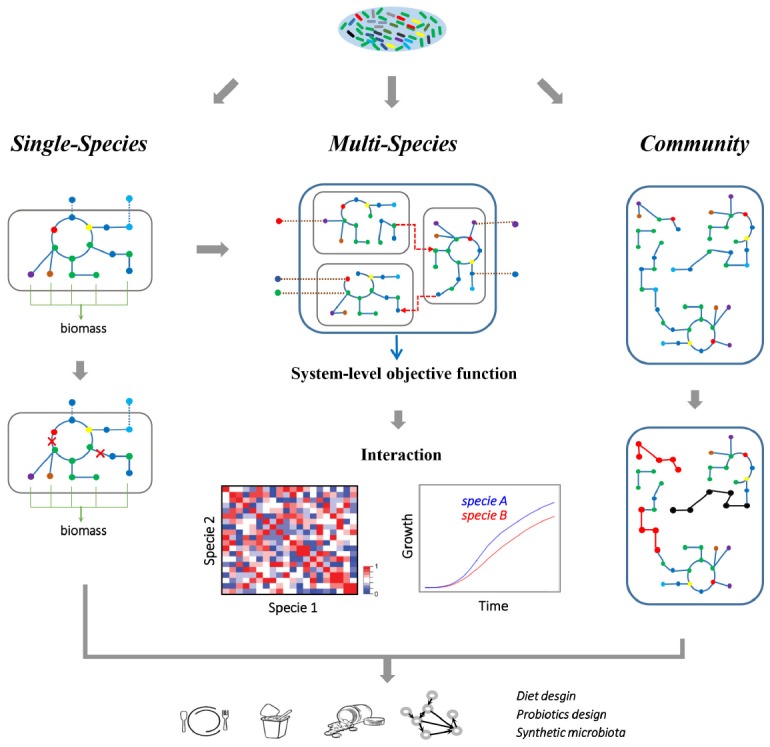
**Metabolic modeling of the gut microbiome.** A single-species GEM is defined as a set of biochemical reactions that occur in a living microorganism, which can be reconstructed starting from the corresponding genome annotation. Here, a single-species model is illustrated, where nodes represent metabolites and edges represent reactions, while the dashed lines indicate exchanges of metabolites between cells and environment. The metabolic capacity and gene essentiality of the single gut species can be inferred using FBA. While multi-species GEMs consider each specie as an individual component, and combine each component with a joint *in silico* environment where the nutrients are supplied. The dashed arrows here indicate the metabolic interactions between different microbial species. With such a multi-component approach, metabolic related phenotype of the whole multi-species system and each species can be simulated. Furthermore, the growth and interaction (cooperation or competition) between microbial species can be inferred at various growth conditions. Alternatively, the community-level metabolic models concentrate on the topology of the metabolic networks, which ignore the specie boundaries and integrate all the metabolic pathways into a community network. Therefore, the topological difference between different models (highlighted with red/black nodes and edges) can be associated with observed differences in metadata, such as healthy and disease states. Altogether, gut microbiome modeling will help in revealing metabolic interactions between microbes or between microbiota and host, and thus provide insight into designing healthy diets, discovering new probiotics and reconstitution of synthetic microbiota.

Genome-scale metabolic models (GEMs) are mathematical representations of the cellular metabolism at the genome level that have served as powerful systems biology tools widely applied for studying microbial metabolism and human health (Oberhardt et al., 2009; Väremo et al., 2013). Historically, the first GEM was developed to study microbial metabolism, starting with the *Haemophilus influenzae* in 1999 (Edwards and Palsson, 1999). Since then, more than 120 GEMs have been reconstructed, but organisms modeled have limited phylogenetic coverages (Monk et al., 2014). Reconstruction of GEMs and the subsequent computational analysis of reconstructed GEMs have been extensively reviewed elsewhere (Thiele and Palsson, 2010; Bordbar et al., 2014). Briefly, metabolic reconstructions are mainly based on the generation of gene-protein-reaction associations inferred from genome annotations and related orthologous information, which link known genes to functional categories and bridge the genotype-phenotype map. Draft reconstructions are typically curated by integrating available information from the literatures, and the reconstructed metabolic network is converted into a stoichiometric matrix where rows represent metabolites and the columns reactions, and thermodynamic and/or physiological constraints can be applied to constrain the feasible space of metabolic operation. Flux balance analysis (FBA) simulates the flow of metabolites through the metabolic network, thereby enabling the use of GEMs for predicting genotype-phenotype relationships of the (Figure 2).

The gut microbiota contributes to the human physiology by its metabolic functions, including energy harvest, bile acid transformations, choline transformation, and the production of short-chain fatty acids (SCFAs), vitamins, and amino acids (Nicholson et al., 2012). Metabolic modeling of the gut microbiome could start from the GEM reconstructions of a few key species from the dominant phyla in the gut ecosystem, and FBA can be applied to explore the metabolic capacities of key gut microbial species. Currently, several gut microbes GEMs have been generated, such as *Bacteroides thetaiotaomicron*, *Eubacterium rectale*, *Methanobrevibacter smithii* (Shoaie et al., 2013), *Bifidobacterium adolescentis* (El-Semman et al., 2014), and *F. prausnitzii* (El-Semman et al., 2014; Heinken et al., 2014). *B. thetaiotaomicron* and *E. rectale* are representatives of the two most abundant phyla in the human gut ecosystem, Bacteroidetes and Firmicutes, respectively. While *F. prausnitzii* is one of the most abundant Firmicutes species in the human gut, and is found to be underrepresented in patients with Crohn’s disease or ulcerative colitis (Sokol et al., 2008). The functional metabolic maps and growth requirements of these beneficial gut bacteria have been extensively explored by simulation with GEMs. *In silico* modeling confirmed the biosynthetic capabilities of SCFAs (acetate, butyrate or propionate) in these species (Shoaie et al., 2013; Heinken et al., 2014). Furthermore, *B. thetaiotaomicron* is able to synthesize all the essential human amino acids from inorganic ammonia (Varel and Bryant, 1974). In contrast, the presence or absence of amino acids in a defined minimum medium significantly affected the production of propionate. Metabolic flux analysis showed that increased fluxes through acetyl-CoA and anaplerotic oxaloacetate synthesis under amino acid deficiency was linked to the overproduction of propionate, which was again accompanied by increased flux through the TCA cycle and reduced regeneration of NAD^+^ through lactate synthesis (Adamberg et al., 2014). Moreover, the GEMs of several probiotic bacteria had been reconstructed, such as *Lactococcus lactis* (Oliveira et al., 2005) and *Lactobacillus plantarum* (Teusink et al., 2006). These GEMs help in identifying metabolites that the bacteria excreted and this facilitated selection and optimization of probiotic strains.

The gut microbiome is a community of microbial species whose metabolism are tightly interacting with each other and with that of the host (Nicholson et al., 2012). Thus, metabolic modeling is not only limited to the study of the gut microbiota at the single-species level, but also extends to the study of metabolic interaction among this multi-species system. Usually, multi-species modeling couples metabolisms of microorganisms in the community by combining single-species models into a joint *in silico* environment where the nutrients are supplied (Figure 2; Stolyar et al., 2007; Taffs et al., 2009). Therefore, this approach considers each organism in the system as an individual compartment where a shared compartment is introduced for the metabolite exchanges between different organisms. The resulting community model can be constrained according to related experimental data, and then be used to infer interactions between components or to predict the phenotype of the whole system in various nutritional conditions. With this multi-component approach, community model of *B. adolescentis* and *F. prausnitzii* GEMs had been used to infer the metabolic interactions between these two gut microbial species (El-Semman et al., 2014). Hereby acetate was found to be a key metabolite exchanged between *B. adolescentis* and *F. prausnitzii*, and the growth and butyrate production of *F. prausnitzii* was found to be dependent on the acetate supply from *B. adolescentis*. Similarly, *E. rectale* also functions as a recipient of acetate. In the presence of *B. thetaiotaomicron*, *E. rectale* takes up acetate produced by *B. thetaiotaomicron* and converts it into butyrate (Shoaie et al., 2013). The main interactions between *B. thetaiotaomicron* and *M. smithii* are the exchanges of acetate and formate. *M. smithii* takes up acetate and formate, and produces methane. With a three-species model (*B. thetaiotaomicron*, *E. rectale*, and *M. smithii*), it was observed that there is competition for acetate between *E. rectale* and *M. smithii*, while CO_2_ and H_2_ produced by *E. rectale* can be taken up by *M. smithii*, and converted into CH_4_ through methanogenesis (Shoaie et al., 2013). Further integrative analyses of transcriptomics data with the GEMs showed that *E. rectale* shifted from polysaccharide utilization to utilization of amino acids, in particular glutamine, in the presence of *B. thetaiotaomicron*. This illustrates how the GEMs could be used to gain new insight into the interactions between species, how there is cross-feeding between them, and how this thus provides novel insights into the commensalism of species within more complex microbial communities. Beyond the representative species of gut microbiota, application of automatic GEM reconstructions starting from thousands annotated gut microbial genome sequences will be helpful for revealing the landscape of the microbe–microbe metabolic interactome (Figure 2). When combining available GEMs with various medium compositions or environmental conditions, the distinct inter-species interactions (neutral, commensal, or mutualistic) and phenotypic properties can hereby be explored, and provide insights into the pattern of metabolic interaction, metabolic potentials of the community, and nutritional scenarios for the gut microbiome.

The gut microbiome is crucial for nutrient acquisition and energy harvest from the diet, and the host–microbe interactions play important roles in the host metabolism (Nicholson et al., 2012). The approach for modeling host–microbe interactions is similar to the way described above for modeling the microbial interactions, and involves the integration of host metabolism and microbial metabolism. Generic human GEMs (Recon 2, HMR 2.0), as well as tissue/cell-specific GEMs (liver, muscle, adipocytes etc.), had been used for host–microbe modeling (Mardinoglu et al., 2013, 2014; Thiele et al., 2013b). One of the host–microbe interaction studies using GEMs was that of Bordbar et al. (2010). The authors inferred the interactions between *Mycobacterium tuberculosis* and human alveolar macrophages by assembling the Mycobacterium GEM into the cytosolic compartment of a macrophage GEM. The integrated host–microbe GEM enabled the simulation of metabolic differences during three infection states, which can act as scaffolds for potential drug target prediction. Moreover, antimalarial drug targets for *Plasmodium falciparum* have been analyzed by integrating a malarial GEM with human erythrocyte or adipocyte GEM (Huthmacher et al., 2010; Bazzani et al., 2012). Recently, such integrative GEM analysis has been applied to characterize the metabolic interactions between the representative gut microbial species *B. thetaiotaomicron* and a mouse on five different diets varying in carbohydrate, fat and protein content (Heinken et al., 2013). FBA revealed that *B. thetaiotaomicron* provides various metabolites to the mouse including essential amino acids, nucleotides, and SCFA (such as acetate and propionate), while the mouse requires the presence of *B. thetaiotaomicron* to synthesize six essential amino acids for optimal growth.

Although the success of using GEMs for recovering metabolic interactions indicates the potentials of these models for gut microbiome research, some significant methodological challenges still need to be addressed. Host–microbe GEMs become more complicated, especially when considering a high number of microbial species in the integrative model. Moreover, multi-species models or host–microbe models that focus on the interior metabolic interactions fail to explain, for example, how variations in species composition affect the metabolic potentials of the microbiome. The limitations of multi-species modeling thus call for different modeling approaches to overcome the interior complexity of multiple components. Alternatively, a comprehensive metabolic modeling approach, treating the entire microbiota as a supra-organism, has been adapted to study the metabolic activity of the gut microbiota as a whole (Figure 2; Taffs et al., 2009; Abubucker et al., 2012; Thiele et al., 2013a). Generally, the community-level metabolic network can be reconstructed directly from shotgun metagenomics data by ignoring cell boundaries and the exchange of metabolites between species (Borenstein, 2012). In an early work (Greenblum et al., 2012), by integrating such microbiome-level metabolic network with corresponding gene abundances, topological differences in both gene-level and network-level were identified as being associated with obesity and IBD. Ultimately, such community-based approaches ignore the boundaries between species and compartmentalization of various metabolites, and provide valuable insight into the metabolic potential and functional divergence of the microbiome in the context of a complete system.

## Gut Microbiome Modeling in Healthcare and Medicine

Diet is one of the major determinants driving the composition and metabolism of the gut microbiome (Scott et al., 2013). Carbohydrates, proteins and fats are the main macronutrients whose amount, type and balance have a great impact on gut microbiota composition and host metabolism. Monosaccharides (i.e., glucose, galactose) are directly absorbed by the intestinal epithelium cells, while numerous dietary polysaccharides, such as resistant starch, non-starch polysaccharides and plant fibers, are able to be digested by the microbes in the gut but not by the human host (Hooper et al., 2002). After carbohydrate fermentation in the proximal colon, proteins are the main energy source in distal colon (Macfarlane et al., 1986). As previously mentioned, *in silico* analysis of integrative bacteria–host GEMs on five different diets varying in fat, carbohydrate, and protein content predicted different growth optima of *B. thetaiotaomicron* and mice (Heinken et al., 2013). The high-carbohydrate diet provides a good carbon source for *B. thetaiotaomicron* and maximizes its growth, which agrees with the fact that *B. thetaiotaomicron* is efficient in utilizing dietary polysaccharides. While the high-protein diet does not support the growth of *B. thetaiotaomicron*, in accordance with the known incapability of *Bacteroides*, to utilize proteins as sole carbon source and the low proteolytic capacity of these bacteria (Heinken et al., 2013). Similar to the prediction, reduced abundance of *Bacteroides* was also observed in the human gut microbiota in subjects having a high-protein/low carbohydrate diet (Duncan et al., 2007). Thus, it is possible to design the diet that modulates the gut microbiota based on the food nutrient composition (Figure 2), which optimizes the growth of the microbiota and benefits human metabolism. Previous applications of GEMs already allow for analysis of the environmental and nutrient requirements of microorganisms, but only restrict it to intensively studied microbes. Consequently, combinations of large-scale GEM reconstructions for gut microbes and media prediction may reveal the possible interaction patterns among gut microbes, and shed light on the nutritional prediction and the design of healthy diets.

Manipulating the gut microbiota with probiotics or prebiotics has been demonstrated to affect the host metabolism (i.e., glucose homeostasis; Rijkers et al., 2010). Probiotic administration with *Lactobacillus* strains have been well characterized regarding potential antimicrobial effects against major gastric and enteric pathogens (Liévin-Le Moal and Servin, 2014). Probiotics and their metabolic products, called postbiotics, have therefore been proposed as food supplements for a healthier intestinal homeostasis and as therapeutic aids for treatment of IBD (Tsilingiri et al., 2012). One major challenge in the development of effective probiotic strains is the limitation of information of the gut microbiome in both healthy and disease states. Although NGS based metagenomics has provided numerous characterizations of genes and species composition, the alterations in the metabolite levels remain unsolved. To overcome this issue, GEM based modeling is an attractive solution due to its predictive ability of microbial metabolism (Figure 2). With GEMs, the biosynthesis of active postbiotics can be systematically explored, which will allow for improved design and optimization of future probiotic strains with enhancing postbiotic production using metabolic engineering. Moreover, the distribution of gut microbiota in the gastrointestinal tract is heterogeneous, which will lead to different prebiotic activities at different gastrointestinal locations (Klemashevich et al., 2014). As demonstrated with the polyphenol quercetin, a probiotic strain may not generate a beneficial effect without cooperative interactions with other strains (Bolca et al., 2013; Klemashevich et al., 2014). Thus, inferring the interactions between gut microbial species via metabolic models, will facilitate the discovery of probiotic mixtures and design of possible biochemical reaction pathways to convert prebiotics into desired postbiotics.

Recently, fecal microbiota transplantation (FMT) has become an alternative treatment compared with standard therapies, and this enables translation of gut microbiota knowledge into clinical use (Borody and Khoruts, 2011). FMT has proved to be effective for treatment of *Clostridium difficile* infections (Bakken et al., 2011). In addition, a long-term follow-up study of FMT in six patients with UC showed reduced symptoms in all patients (Brandt et al., 2012). Usually, FMT involves transplantation of fecal bacteria from a healthy individual into a recipient, which hereby has gut microbiota restored by a healthy bacterial flora. Nevertheless, concerns about pathogen transmission, patient acceptance and treatment standardization still remain (Claes et al., 2015), and FMT is therefore mainly used for patients where there are no alternative treatment options. For example, two patients with recurrent *C. difficile* infection that was unresponsive to conventional therapy, were cured by transplantation of a synthetic microbiota composing of 33 bacterial cultures isolated from the feces of a healthy donor (Petrof et al., 2013). Recently, a rational design of microbiota including six phylogenetically diverse intestinal bacteria cleared *C. difficile* infection in mice and restored the healthy microbiota (Lawley et al., 2012). Consequently, the next step would be to use gut microbiome knowledge to improve human health by designing ideal synthetic microbiota for FMT. Therefore, a bottom-up approach integrating GEMs of single species with pre-defined functions will allow for identification of the interaction patterns between multi-species and result in possible species combinations with desirable metabolic functions (Figure 2). As mentioned above, the interaction between two microbial species can be neutral, commensal, or mutual (Thiele et al., 2013a). Mapping all possible interaction patterns and identification of all possible metabolite exchanges between two species will drive the assembly of complex microbiota by maximizing paired cooperation. Moreover, by application of metabolic engineering tools and the rational design of microbiota, it will be possible to simplify the complexity of synthetic microbiota, and provide fundamental knowledge that can be used to infer intrinsic mechanisms of how microbiome influences human health.

## Future: the Nexus of Systematic Modeling and the Gut Microbiome

With the advent of NGS-based metagenomics and development of related bioinformatics approaches, we have gained a deep understanding of the gut microbiome and its impact on human disease and health. Such sequencing based surveys focus on the taxonomic or functional compositions of the gut microbiome, but provide little information about the mechanism of metabolic interactions. Therefore, to reveal the underlying metabolic principle of host–microbe interactions or microbe–microbe interactions, it is necessary to go beyond solely characterizing the gut microbiome composition and toward systematic modeling and analysis of the gut microbiome (Hanage, 2014; Waldor et al., 2015). The application of metabolic modeling approaches to gut metabolic interactions is therefore a critical next step in gut microbiome studies. Such system-level metabolic reconstruction provides a predictive understanding of the metabolic capacities in the gut microbial species and community, and associates the metabolic changes with the disease or healthy states. Moreover, the GEM based modeling approach is helpful for systematical analysis of the gut microbiome through the quantitative integration of transcriptomics, proteomics and metabolomics data with metabolic phenotypes. Finally, GEMs based *in silico* growth or metabolite production prediction can be easily compared with obtained experimental data to provide theoretical explanation for observed metabolic phenotypes.

Due to the inherent complexity and heterogeneity of the gut microbiome, a simple approach to study the metabolic interactions is to build artificial microbiota from monocultures in defined combinations (Mee and Wang, 2012; Vos, 2013). Therefore, there is a clear need to further develop both the experimental and design framework for synthetic microbial communities. GEMs have been successfully applied to develop cross-feeding microbial communities for industry (Wintermute and Silver, 2010). In these engineered microbial communities, syntrophic growth can be achieved by exchanging cross-feeding metabolites across different species. Similarly, the principle of syntrophic design can be applied to develop engineered probiotics with enhanced catabolism of nutrients or biosynthesis of postbiotics. In addition, the application of modeling approach requires systematic manipulation of gut microbiota through well-designed *in vitro*/*in vivo* experiments for *in silico* model testing and validation. An example of such an *in vitro* system is the simulator of the human intestinal microbial ecosystem (SHIME; Van den Abbeele et al., 2010). This system mimicked the fermentative processes in the stomach, small intestine and three colon regions, and supported anaerobic growths of the microbiota. Combining such *in vitro* co-culture system with predefined gut microbiota will allow a controlled testing system for engineered microbial consortia. Similar to *in vitro* systems, germ-free (GF) animals, when colonized with synthetic microbial consortia, will be able to associate specific functions or specific microorganisms with the host, and thus be able to validate interactions between pre-defined microbiota and the host (Karlsson et al., 2013a). Furthermore, the alteration of diet, environment, or genetic background can be integrated into these *in vivo*/*in vitro* experimental systems to simulate the host–microbiota or microbe–microbe interactions. Finally, metabolic modeling combined with knowledge and data from experiment will greatly strengthen our understanding of metabolic interactions among microbes or between the microbe and host, and hereby provide insight into the clinical application of gut microbiota in diagnoses and therapies.

### Conflict of Interest Statement

The authors declare that the research was conducted in the absence of any commercial or financial relationships that could be construed as a potential conflict of interest.
